# Single cell quantification of microRNA from small numbers of non-invasively sampled primary human cells

**DOI:** 10.1038/s42003-023-04845-8

**Published:** 2023-04-26

**Authors:** Vanessa Ho, Jonathan R. Baker, Keith R. Willison, Peter J. Barnes, Louise E. Donnelly, David R. Klug

**Affiliations:** 1 Institute of Chemical Biology, Molecular Sciences Research Hub, Imperial College London, 82 Wood Lane, White City, London, W12 0BZ UK; 2 Department of Chemistry, Molecular Sciences Research Hub, Imperial College London, 82 Wood Lane, White City, London, W12 0BZ UK; 3 National Heart and Lung Institute, Imperial College London, Guy Scadding Building, Dovehouse Street, London, SW3 6LY UK

**Keywords:** Assay systems, Prognostic markers

## Abstract

Expression levels of microRNAs (miRNAs) in single cells are low and conventional miRNA detection methods require amplification that can be complex, time-consuming, costly and may bias results. Single cell microfluidic platforms have been developed; however, current approaches are unable to absolutely quantify single miRNA molecules expressed in single cells. Herein, we present an amplification-free sandwich hybridisation assay to detect single miRNA molecules in single cells using a microfluidic platform that optically traps and lyses individual cells. Absolute quantification of miR-21 and miR-34a molecules was achieved at a single cell level in human cell lines and validated using real-time qPCR. The sensitivity of the assay was demonstrated by quantifying single miRNA molecules in nasal epithelial cells and CD3^+^ T-cells, as well as nasal fluid collected non-invasively from healthy individuals. This platform requires ~50 cells or ~30 µL biofluid and can be extended for other miRNA targets therefore it could monitor miRNA levels in disease progression or clinical studies.

## Abstract (web summary)

An amplification-free single-cell microRNA assay with single molecule sensitivity allows quantification of miRNA in human nasal fluid collected non-invasively, demonstrating its potential utility in quantitively monitoring miRNA levels in clinical studies.

## Introduction

MicroRNAs (miRNAs) are short (~22 nucleotides) single-stranded RNAs that play a role in cellular processes including differentiation, proliferation, and apoptosis^1–3^ MiRNAs regulate post-transcriptional gene expression with each miRNA having the capacity to suppress many mRNA molecules^3^. However, dysregulation of miRNA expression is associated with many disease processes^2,4^. Conventional methods to detect miRNA such as Northern blotting^5,6^, real-time qPCR^7,8^, next-generation sequencing (NGS)^9^ and microarray^10,11^ usually require large numbers of cells. In addition, the output often represents the average ensemble expression of the cell population that will not reflect cellular heterogeneity^12^ and expression of specific miRNA molecules in rare cell subsets may not be identified. Therefore, there is a requirement for measurement of miRNAs at a single-cell resolution in order to understand their regulation and function more specifically.

Microfluidic technology allows single-cell analysis to be performed at relatively low cost, with rapid readouts, high-throughput and reduced sample and reagent consumption^13,14^. Multiple microfluidic-based cell isolation techniques have been developed such as microstructural trapping/encapsulation^13,15,16^ or external force-mediated trapping^17,18^. These techniques can then be coupled with analytical techniques such as fluorescence^19,20^, Raman spectroscopy^21^ and mass spectroscopy^22,23^. Microfluidic systems can encapsulate single cells into droplets^24–27^. The relative abundance of miRNA in a single cell can be low with some cells expressing as few as 10^2^ miRNA copies^28^. As such, nucleic acid amplification such as PCR amplification^29^, rolling circle amplification (RCA)^30,31^ are often used to improve the sensitivity and selectivity of miRNA detection in isolated single cells^30,32^. However, this requires complex sequence design and may lead to false-positive/negative results due to the similarities in miRNA sequences^32^.

For microarray analysis of miRNAs, enzymatic or direct chemical labelling of miRNAs are commonly used for hybridisation with capture probes, which is costly and time consuming^33–35^. Direct miRNA detection methods have been developed, such as nano-pore^36,37^, nano-wire^38,39^, electrochemical^40,41^, surface plasmon resonance (SPR)^42,43^ and surface enhanced Raman spectroscopy (SERS)^44^. The plasmonic affinity sandwich assay (PASA) detects miRNAs at a subcellular level through labelling extracted target miRNA with Raman nanotags composed of silver nanoparticles^45^. This was further developed to quantify circulating miRNAs by using gold nanoparticles decorated with silver nanocomposites^46^. Although these methods are highly sensitive, they require expensive and complex instrumentation, and may not be easily adaptable for multiplexing.

An alternative label and amplification-free method to detect miRNAs is designing a sandwich hybridisation assay involving capture and fluorescently tagged reporter probes. This approach increases the specificity of detecting miRNAs as it relies on the hybridisation events of the target miRNA with complimentary oligonucleotide probes. Different sandwich microarray platforms have been developed to detect miRNAs in fluids^35,47,48^. The coaxial stacking effect can aid sandwich hybridisation of oligonucleotides and miRNA within a microenvironment and provide a rapid readout^49^. While there are ongoing developments in microfluidic approaches for the quantification of miRNA in single cells, there is no current single microfluidic device that enables single-cell isolation and lysis, and detection of the released single miRNA molecules without labelling or amplification requirements.

We previously developed a microfluidic platform termed ‘microfluidic antibody capture’ (MAC) to optically isolate and lyse single cells to absolutely quantify protein expression with a high precision and dynamic range^50–53^. In the present study, the MAC chip technology has been adapted for detection and quantification of miRNA molecules in single cells. This assay utilises sandwich hybridisation to capture target miRNAs from a single cell that are detected using a complementary sequence labelled with a fluorescent reporter probes and total internal reflection fluorescence (TIRF) microscopy.

To develop this system, we examined the expression of two miRNA that are known to be expressed in several cell types, for example miR-21^54^. We also examined a second miRNA, miR-34a that is expressed in epithelial cells^55^. We were able to show that this technique allows amplification-free, absolute quantification of single miRNA molecules in single epithelial cells and CD3^+^ T-cells. In addition, we further used this system to measure miRNA in human nasal fluid collected non-invasively^56^. Our results demonstrate that this is a highly sensitive and amplification-free microfluidic platform to quantify miRNAs in cells and biofluids that may be of use to quantitively monitor miRNA levels in clinical studies.

## Results

### Assay development

**Fig. 1:**
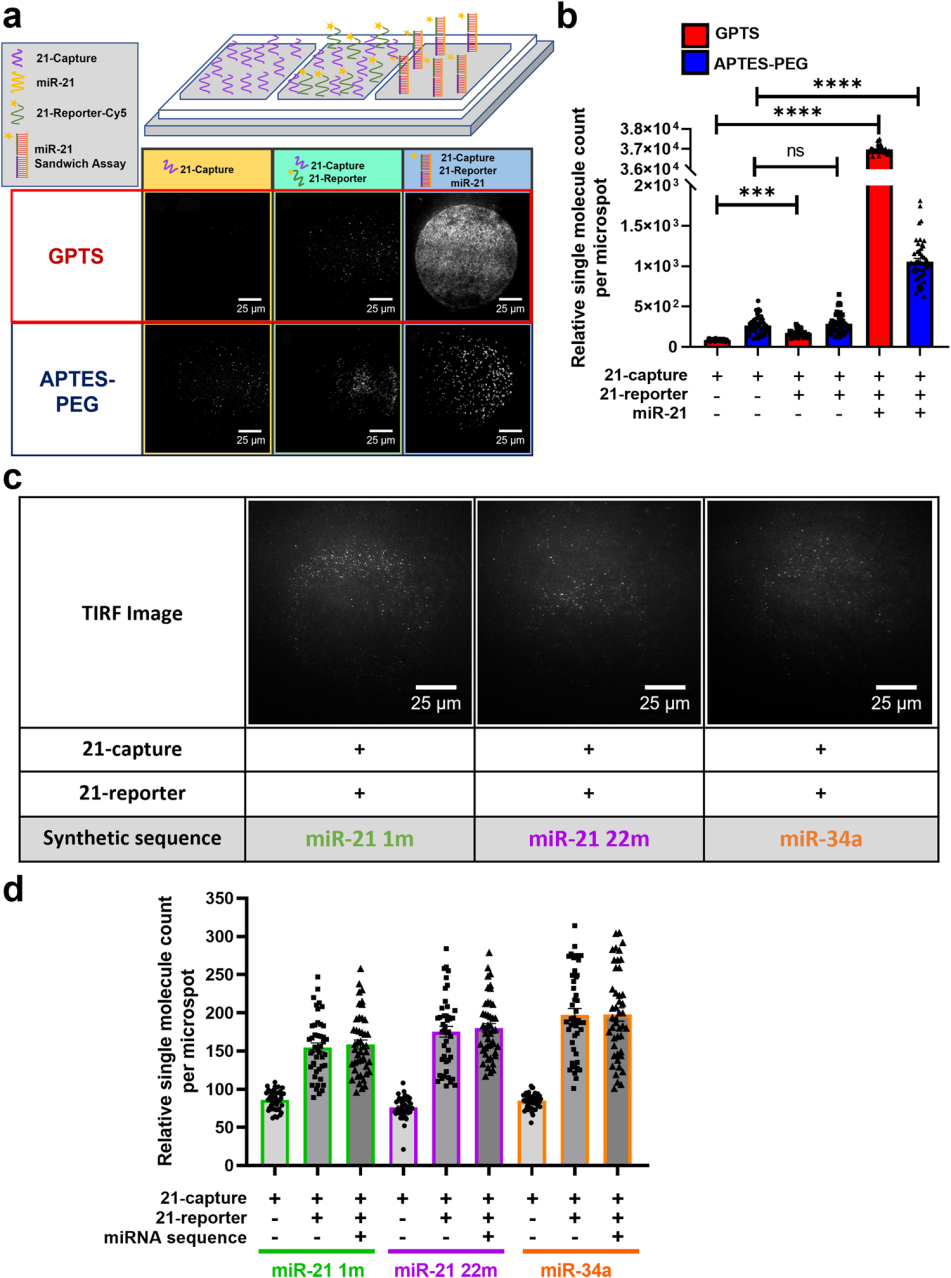
MiR-21 assay development. **a** Schematic diagram of an open chip platform consisting of three wells comparing GPTS (Red) and APTES-PEG (Blue) passivated surfaces: first well with 21-capture probe, second well with 21-capture and 21-reporter probes, and third well containing 1.2 ×10^6^ molecules/nL miR-21 sequences with 21-capture and 21-reporter probes. TIRF images show single molecule binding events taking place on GPTS and APTES-PEG functionalised surface within the open chip. **b** Bar graph of single miR-21 molecules detected using the 21-capture microarrayed spots within the open chip. Open chips were incubated and imaged every 10 min for 2 h. Data are presented as individual points and mean ± SEM analysed by Kruskal-Wallis test with post hoc Dunns ***p < 0.001 and ****p < 0.0001. **c** Raw TIRF images of microarrayed spots showing negligible single molecule binding of 21-capture and 21-reporter with miR-21 single-base modification at position 1 (miR-21 1m), 22 (miR-21 22m) and miR-34a. **d** Bar graph of relative single molecule count detected on 21-capture microarrayed spots in an open chip device with miR-21 1 m (green), mir-21 22m (purple) and miR-34a (orange) sequences present in individual wells. Open chips were incubated and imaged every 10 min for 2 h. Data are presented as individual points and mean ± SEM.

An open chip platform consisting of three wells was used to perform preliminary experiments (Supplementary Fig. 1). Chemical surface passivation was performed to minimise non-specific interactions and two common chemical passivation silanes for glass surfaces, aminopropyltriethoxysilane polyethylene glycol (APTES-PEG) and glycidoxypropyltrimethoxysilane (GPTS) were compared (Fig. 1a and Supplementary Fig. 2)^51,57,58^. Homogenous miR-21-capture spots were microarray printed onto chemically functionalised surface within each well of the open chip. For both surfaces, a low level of non-specific interaction with miR-21 capture was observed in the well that contained only hybridisation buffer (Fig. 1a). However, less non-specific binding events were observed between miR-21-capture probe and GPTS surface, 85.9 ± 2.2 relative single molecules, compared to biotinylated 21-capture probe and APTES-PEG surface, 266.6 ± 16.2 relative single molecules (Fig. 1b). While oligonucleotide-oligonucleotide crosstalk between miR-21 capture and reporter probes was observed for both surfaces in presence of hybridisation buffer (Fig. 1a), GTPS surface revealed less crosstalk between capture and reporter probes, 171.8 ± 7.5 relative single molecules compared to APTES-PEG surface, 284.5 ± 17.9 relative single molecules (Fig. 1a, b). This could be due to the strong amide bond formation between the amino group of the capture probe and the layer of epoxide functional groups on the glass surface (Supplementary Fig. 2). Synthetic miR-21-5p sequences were then introduced into the third well to ensure that miR-21 sequences were being captured and detected by the probes (Fig. 1a, b). The relative single miR-21 molecules captured on GPTS surface was 3.7 × 10^4^ ± 24.9 with a capture efficiency of 82.2%, which is an order of magnitude greater compared to APTES-PEG passivated surface with relative single molecule count of 1.06×10^3^ ± 40.3 and a capture efficiency of 2.35% (*n* = 3) (Fig. 1b). From these experiments, GPTS passivated surface was concluded to provide a lower background signal, higher signal to noise ratio and greater capture efficiency compared to APTES-PEG surface. Therefore, future experiments were conducted with GPTS passivated surface to increase the sensitivity of the miRNA assay.

### Assay specificity

MiRNA strands are short sequences of 16-28 nucleotides so achieving a high specificity for miRNA assays can be challenging due similar miRNA sequences that can potentially bind to the capture probe^59–61^. The specificity of the miRNA assay is enhanced because both complimentary probes are required to hybridise with target miRNA in order to detect single miRNA molecules using TIRF microscopy, which only detect molecules that are bound onto surface within the evanescent field, as shown in Fig. 1a.

The specificity of the miR-21 assay was determined by introducing synthetic one single-base mismatched miR-21 sequence and non-complimentary miR-34a sequence into separate open chips. MiR-21 1m and miR-21 22m are modified miR-21 sequences that contained mismatched base at position 1 and 22 (5’–3’), respectively. The relative single molecule count compared to control, without target miR-21 sequences, increased by 2.46% in presence of miR-21 1m sequence, 2.74% with the miR-21 22m sequence and 0.15% with the miR-34a sequence (Fig. 1c, d). The presence of these sequences has a negligible effect on the single molecule count, suggesting that the miR-21 assay has a high specificity and high hybridisation efficiency in capturing miR-21 sequences.

### Assay optimisation

Previous studies have shown a higher miRNA heterogeneity at the 3’ compared to the 5’ end of the sequence^62,63^. MiRNA isoforms are usually identical at the 5’ end and differ at the 3’ end and isoforms are assumed to bind to the same target mRNA with a different degree of target repression^3^. For this reason, the microarrayed capture probes were designed to hybridise with target miRNA from the 3’ end. The capture probe was sequenced with oligonucleotide d(T) 12 bases and C12 spacer because this length of spacers was previously reported to result in the highest hybridisation yield and signal on immobilised surface^35^. To maximise the capture and hybridisation efficiencies of the miRNA assays, different concentrations of the oligonucleotide probes were tested using the open chip platform surface passivated with GPTS (Supplementary Fig. 3).

Open chips consisting of three wells were used to optimise the capture probe concentration, whereby each well was microarrayed with 8 rows of miRNA 21-capture at concentrations ranging from 10^3^–10^9^ molecules/nL for simultaneous detection (Supplementary Fig. 3). GPTS passivated coverslips were microarray printed with capture probes on the same print run to ensure reproducibility of micro spot morphologies. Similarly, open chips with 6 wells were utilised to determine the optimum concentration of the miRNA 21-reporter probe. Within each well, three rows of 1.0 × 10^8^ molecules/nL miRNA 21-capture were microarrayed and different concentrations of miRNA 21-reporter 10^7^–10^11^ molecules/nL were introduced into corresponding well (Supplementary Fig. 3).

The concentration of miRNA 21-capture molecules microarray printed on the surface directly influences the number of miR-21 molecules that can be captured and detected. This can limit the maximum number of specific binding events of that can occur and thereby limiting the sensitivity and dynamic range of the assay. From these experiments, the optimum concentration of miRNA 21-capture was 1.0 × 10^8^ molecules/nL, with a capture efficiency of 84.6% in the presence of 1.2 × 10^6^ synthetic miR-21 molecules/nL. The optimum concentration of 21-reporter probe was 6.02 × 10^9^ molecules/nL, with a hybridisation efficiency of 91.4%.

### Assay calibration

**Fig. 2:**
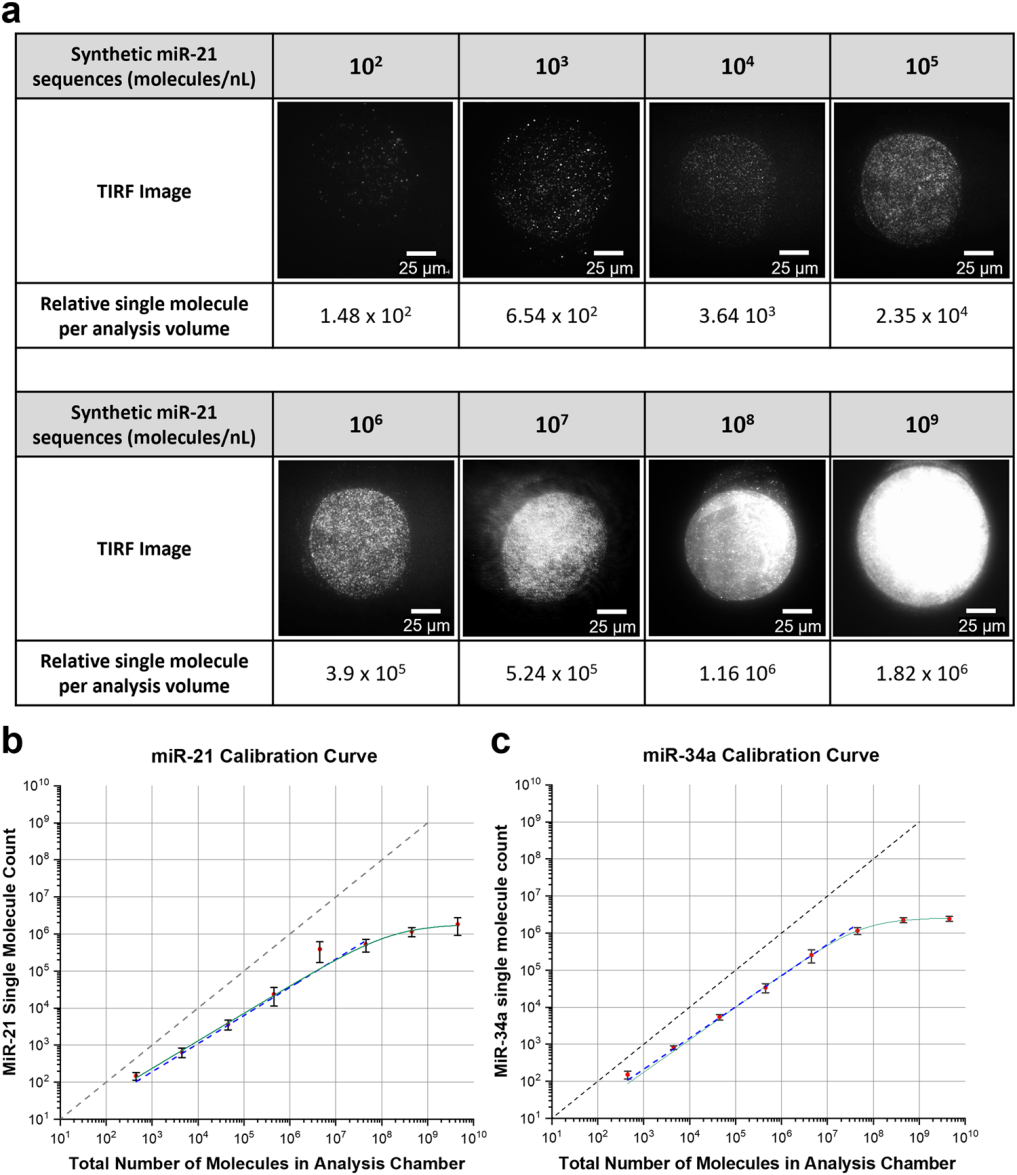
MiR-21 and miR-34a assay calibration using microfluidic chip with 4.5 nL chambers. **a** Raw TIRF images of 21-capture microarrayed spots within an analysis chamber containing different concentration of synthetic miR-21. Calibration curves were fitted using a non-linear Hill function shown in green and a power function was plotted in a dashed blue line. Black line represents 100% capture efficiency. Calibration for **b** miR-21 assay and **c** and miRNA-34a assay. Data are presented as mean ± SEM.

To quantify miR-21 and miR-34 molecules expressed in a single cell within the analysis chamber, calibration curves were generated to determine the dynamic range of the miRNA assays. Known concentration of synthetic target miRNA was introduced into a series of microfluidic chips with 4.5 nL chambers. Batches of microfluidic chips were fabricated and individually filled with varying concentrations of target miRNA sequences 10^2^–10^9^ molecules/nL prepared with reporter probe and hybridisation buffer. The calibration was conducted in this manner to avoid differences between the assay microenvironments during generation of the calibration curve or a single-cell experiment. A calibration curve was plotted to enable absolute number of target miRNA molecules to be extrapolated from the relative single molecule counts obtained from TIRF images (Fig. 2a). This resulted in a linear range of 4.5 × 10^2^ to 4.5 × 10^7^ molecules/nL. The lower limit of detection for both miR-21 and miR-34a assay was determined to be 4.5 × 10^2^ molecules/nL (Fig. 2b, c). The background molecular count for miR-21 and miR-34a assay was 76 ± 2.45 and 90 ± 3.77 molecules/nL (*n* = 3), respectively. The batch-to-batch variation for miR-21 and miR-34a assay microfluidic chip fabrication was 2.7% and 3.3%, respectively.

### MiRNA absolute quantification in cell lines

**Fig. 3:**
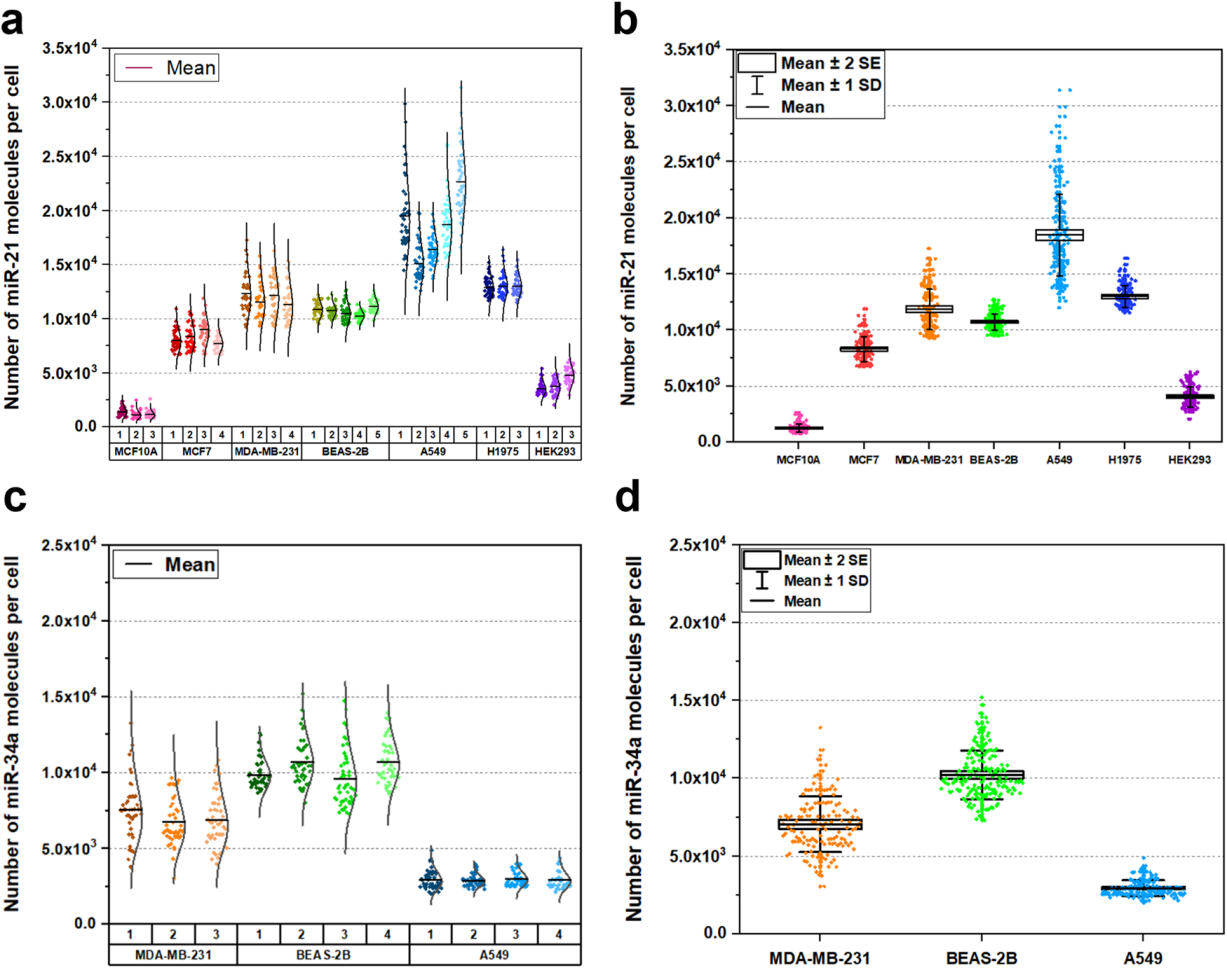
Distribution of single miR-21 and miR-34a molecules expressed at a single-cell level in various cell lines. Relative single molecule count obtained from raw TIRF images were converted into absolute number of miR-21 or miR-34a molecules/cell using the corresponding calibration curves. Each data point represents the number of miRNA molecules expressed in one cell. Experimental repeats (*n*) for the same population of cells are labelled 1–5 (**a** and **d**). The black solid line indicates the mean. **a** Baseline expression of miR-21 in (from left to right) MCF10A (Pink; *n* = 3), MCF7 (Red; *n* = 4), MDA-MB-231 (Orange; *n* = 4), BEAS-2B (Green; *n* = 5), A549 (Light blue; *n* = 5), H1975 (Navy; *n* = 3) and HEK293 (Purple; *n* = 3) cells using single-cell analysis. **b** Box and whisker plot for miR-21 expressed in each cell line. **c** Baseline expression of miR-34a in MDA-MB-231 (Orange; n = 3), BEAS-2B (Green; *n* = 4), A549 (Light blue; *n* = 4) cells using single-cell analysis. **d** Box and whisker plot for miR-34a expressed in each cell line. Normal or gamma distribution curves were used to fit the data to highlight the shape of each distribution.

Having established an assay system, next the assay was tested to determine whether this could be used to quantify single miRNA molecules in single cells. Single-cell experiments (Supplementary Fig. 4) were then performed on several different human cell lines including bronchial epithelial BEAS-2B cells, lung adenocarcinoma (A549) cells, non-small cell lung cancer epithelial (H1975) cells, breast epithelial (MCF10A) cells, breast adenocarcinoma (MCF7 and MDA-MB-231) cells, and embryonic kidney epithelial HEK293 cells. Experimental repeats performed on the same cell line were grown in separate culture flasks seeded with the same cell density and passage number. Relative single molecule count obtained from TIRF images were converted into absolute number of miR-21 or miR-34a molecules using the calibration curves (Fig. 2b, c). Expression of single miR-21 molecules were successfully captured, detected and quantified in seven human epithelial cell lines at a single-cell resolution (Fig. 3a, b). Similarly, single miR-34a molecule expression in single cells were detected and quantified in three human epithelial cell lines (Fig. 3c, d).

The differences in abundance of miR-21 in cell lines varied markedly (Fig. 3a, b). MiR-21 is one of the first oncomiRs discovered and is upregulated in several cancers including breast and lung cancer^54,64–66^. The distribution of miR-21 expression in human breast epithelial cells, MCF7 was 8.28×10^3^ ± 70.9 molecules/cell (*n* = 4) and MDA-MB-231 was 1.18×10^4^ ± 62.3 molecules/cell (*n* = 4), whereas in the adenocarcinoma human alveolar epithelial cell line, A549 was 1.85×10^4^ ± 390 molecules/cell (*n* = 5) were detected. These cells showed the greatest heterogeneity compared to non-cancerous breast cells, MCF10A 1.24 × 10^3^ ± 19.8 molecules/cell (*n* = 3) and the bronchial epithelial cells, BEAS-2B, 1.09 × 10^4^ ± 46.0 molecules/cell (*n* = 5) (Fig. 3b). A long tail distribution was observed for MCF-7, MDA-MB-231 and A549 cell lines as the outlier cells expressed greater number of miR-21 molecules than the mean of the cell population. This heterogenity in miRNA expression is a common characteristic of cancerous cells^67,68^, and expression of miR-21 was greater in MCF-7 and MDA-MB-231 cells compared to MCF-10A cells, which agrees with previous studies^27,64,69^. The abundance of miR-21 in MDA-MB-231 cells is higher than in MCF-7 cells, this observation is also consistent with previous studies^27,69^. These data demonstrate that the miR-21 assay can be used to quantify single miR-21 molecules in single cells requiring ~50 cells per experiment.

Similarly, the variation in absolute number of miR-34a molecules in single cells can be compared between the cell lines (Fig. 3c, d). Notably, the distribution of miR-34a expression in A549 cell line is narrower compared to other cell lines (Fig. 3d). The coefficient of variation for miR-21 assay and miR-34a in BEAS-2B cells is 2.88% and 5.87%, respectively. This indicates that BEAS-2B cell line is an appropriate cell line to use for cell transfection.

### Validation of miRNA assays

To further validate the miR-21 and miR-34a assays, BEAS-2B cells were transfected with specific miRNA mimics or an antagomirs for 24 h. Single-cell experiments require ~50 cells and were performed in parallel with bulk cell analysis real-time qPCR that require ~10^6^ cells. BEAS-2B cells were chemically lysed for real-time qPCR at the same time point as cells used to perform single-cell experiments (Supplementary Fig. 5).

**Fig. 4:**
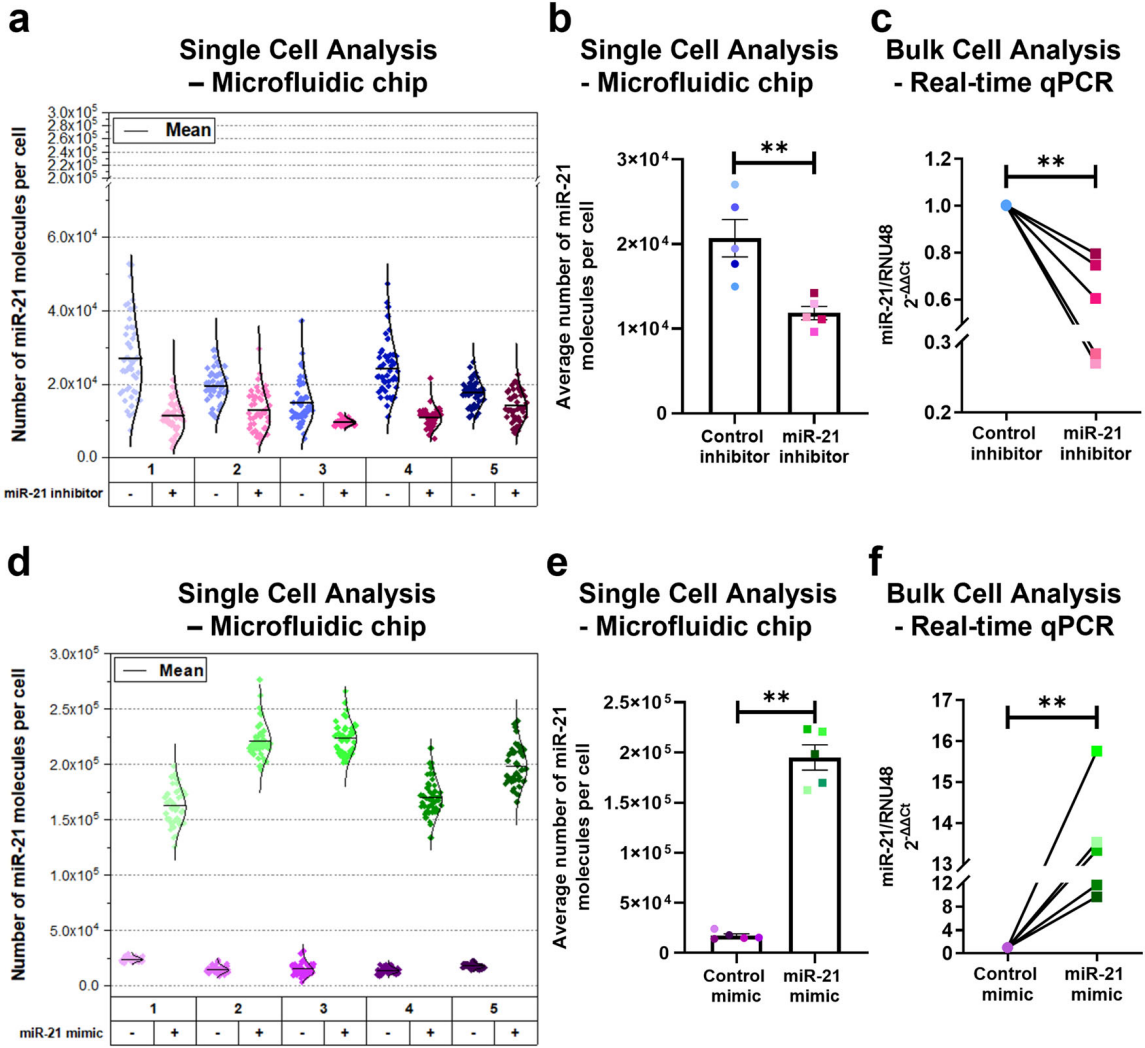
Validation of miR-21 assay using specific miR-21 mimics and antagomirs. BEAS-2B cells were overexpressed with miR-21 mimic (Green), miR-21 inhibitor (Pink), control mimic (Blue) or control inhibitor (Purple) for 24 h and the expression of miR-21 was assessed using microfluidic platform and real-time qPCR (n = 5). The same shade of colour represents the same population of BEAS-2B cells. The different shade of colour represents experimental repeats, labelled 1-5 (**a** and **d**). **a** Single-cell distribution of miR-21 molecules expressed in single BEAS-2B cells transfected with inhibitor or control. **b** Comparison of average miR-21 expression between BEAS-2B cells transfected with inhibitor and control measured by single-cell analysis. **c** Relative miR-21 expression (ΔΔCT) in BEAS-2B cells transfected with inhibitor or control measured by real-time qPCR, normalised to RNU-48 and its control. **d** Distribution of miR-21 expression in BEAS-2B cells transfected with mimic or control measured in single cells. **e** Comparison of average miR-21 expression in single BEAS-2B cells transfected with mimic or control. **f** Relative miR-21 expression (ΔΔCT) in BEAS-2B cells transfected with mimic or control by real-time qPCR, normalised to RNU-48 and its control. The black solid line represents a normal or gamma distribution fit to the data and the mean. Data were analysed using Mann–Whitney *U*-test, **p < 0.01.

In single-cell analysis, BEAS-2B cells that had been transfected with a miR-21 antagomir showed significantly decreased miR-21 expression by ~58% and conversely cells transfected with a miR-21 mimic significantly increased miR-21 expression by 11-fold (Fig. 4a, b, d and e). These changes are consistent with real-time qPCR data (Fig. 4c, f). A similar pattern was observed when BEAS-2B cells were transfected with an antagomir of miR-34a, which showed a reduced miR-34a expression by 27% in single-cell analysis. Whereas in BEAS-2B cells that were transfected with a miR-34a mimic, there was enhanced expression of miR-34a by 25-fold (Supplementary Fig. 6a, b, d and e). Again, these data, were comparable with real-time qPCR analysis (Supplementary Fig. 6c, f).

### Nasal samples

The purpose of establishing this microfluidic technique was to devise a methodology with the capacity to measure single-cell miRNA expression in samples where cells are scarce. Nasal sampling is one such technique and if the assay could be applied to these samples, it is possible that this technique would have wider applications in clinical research. To test this, nasal samples were collected non-invasively from 6 volunteers using SAM to establish a preliminary healthy baseline level of miR-21. The collects nasal samples were separated into nasal cells and fluid to perform single miR-21 molecule detection on separate microfluidic chip devices using the workflow highlighted in (Supplementary Fig. 7).

**Fig. 5:**
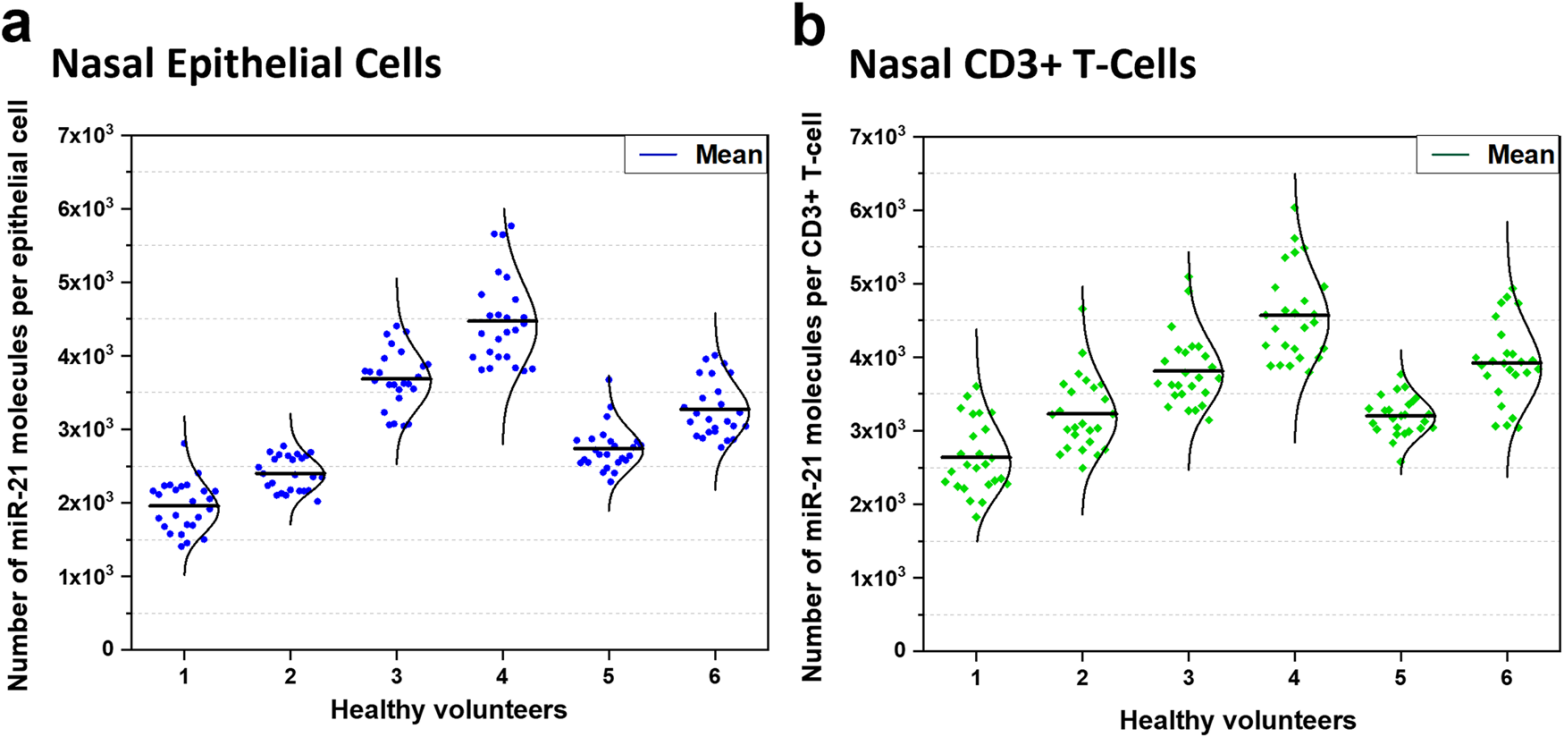
Baseline expression of miR-21 in nasal epithelial cells and CD3+T-cells collected from six healthy volunteers. Nasal cells were collected using SAM. Nasal fluid and cells were separated using centrifugation. On the same day of collection, single-cell analysis was performed to quantify levels of miR-21 in cells. For each volunteer, nasal cell sample was analysed on separate a microfluidic chip. EpCAM^+^ epithelial cells (*ʎ* = 488 nm) and CD3^+^ T-cells (*ʎ* = 532 nm) were isolated into individual micro-chambers. Relative single molecule count detected from raw TIRF images were converted into absolute number of miRNA using the corresponding calibration curves. Distribution of miR-21 molecules expressed in **a** epithelial cells (Blue; 25 cells) and **b** CD3^+^ T-cells (Green; 25 cells) at a single-cell level for each volunteer (1–6). Normal or gamma distribution curves were used to fit the data to highlight the shape of each distribution with the solid black line representing the mean.

**Fig. 6:**
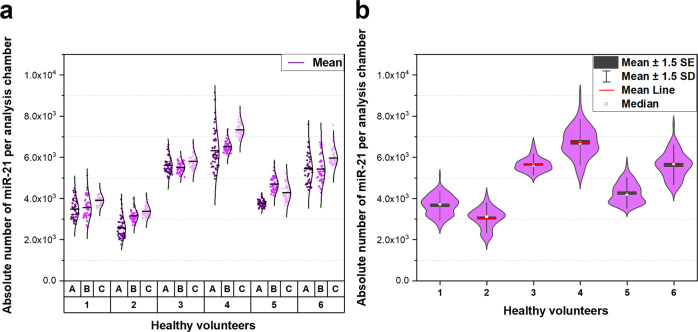
Baseline expression of miR-21 was quantified in nasal fluid collected non-invasively from six healthy volunteers. Nasal samples were collected using SAM. Nasal fluid and cells was separated using centrifugation. Six volunteers (1–6) were sampled three times (A–C) within 7 days. Each nasal fluid sample was analysed on separate microfluidic chips on the same day of sample collection. 30 µL of nasal fluid was introduced into the microchannel, miR-21 molecules were diffused from the channel into analysis chambers for single molecule detection. Relative single molecule count of miR-21 was quantified using miR-21 calibration curve. **a** Distribution of miR-21 molecules expressed in each nasal fluid sample. The different shades of purple represent three different nasal fluid samples obtained from the same individual (A–C). **b** Violin plots comparing miR-21 expressed in nasal fluid collected by different healthy volunteers. The box represents the SEM, and the whiskers represent 1.5x SD. Each box has the rotated kernel density plot on either side. Normal or gamma distribution curves were used to fit the data to highlight the shape of each distribution.

Epithelial cells (EpCAM^+^) and T-cells (CD3^+^) were identified using antibodies to label cell surface markers and imaged using TIRF microscopy at wavelengths 488 and 532 nm, respectively (Supplementary Fig. 6). Within the microfluidic chip device, these target cells were manoeuvred using optical tweezer from the microchannel into individual chambers and then optically lysed. Using this technique, baseline expression of miR-21 was quantified in epithelial cells (3087 ± 833 molecules/cell) and CD3^+^ T cells (3561 ± 619 molecules/cell; *n* = 6) (Fig. 5a, b). The distributions shown in Fig. 5a, b, reveals the cellular heterogeneity in nasal epithelial cells and CD3^+^ T-cells, not only with cell type but also with each individual. Having shown that this technique could be used to measure small numbers of cells (~50 cells), it was possible that cell free miRNA, either free or contained in extracellular vesicles, may also be detected in nasal fluid. To assess this, a small volume (~30 µL) of nasal fluid, was introduced into microchannel of the chip and the average expression of miR-21 in nasal fluid determined as 4130 ± 1278 molecules/nL (*n* = 6). To see if these observations were reproducible, nasal fluid samples were also collected from each volunteer three times within 7 days. The widths of the distribution of miR-21 expression varied, displaying day-to-day variability for each volunteer (Fig. 6a). Most of the nasal fluid sample show a symmetrical Gamma distribution (Fig. 6a). The changes in the shape of the distribution within each volunteer is more apparent shown in violin plots in Fig. 6b, where the mean and median values can be comparable. The coefficient of variation between healthy volunteers is 2.19–11.22% over these times points. Nevertheless, this demonstrates the robustness of miR-21 assay developed is capable to quantify small volumes of biofluid and different cell types.

## Discussion

Single-cell miRNA detection techniques are improving; however, these methods still rely on nucleic acid or signal amplification, which increases experimental time, costs and potentially lead to bias results. Alternatively, label-free miRNA sandwich assays have been developed to quantify miRNA in human samples but at present, lack single miRNA molecule sensitivity in single cells^38,39,48^. In this study, we developed a sandwich hybridisation-based assay using a miniaturised MAC chip, device to absolutely quantify miRNA in small number of cells or volume of biofluids where there is a low abundance of target miRNA. The advantage of combining microfluidics and microarray technologies reduces the cost and hybridisation time. Our method encompasses a ‘lab-on-a-chip’ where we isolate and lyse cells and detect specific miRNA on a single chip. However, there are limitations to this approach. For instance, at present, each cell must be manually isolated using an optical tweezer. This is time consuming and takes up to 25–30 min to isolate 50 cells. However, this limitation can be addressed in the future by automation and motion control mechanics. There is also a design requirement for complimentary capture and reporter probes to hybridise to the target miRNA. Finally, there is a requirement for calibration to allow absolute quantification of miRNA that may limit sensitivity.

The chemical surface passivation was investigated initially, the capture efficiency was greater for GPTS surface compared to APTES-PEG, due to the formation of strong amide bond between the epoxide ring on the glass surface and amine group on the oligonucleotide. MiRNA assays were designed with a capture and reporter probe that are complimentary to the target miRNA. The developed miRNA assays were sensitive to capture and quantify target miRNA levels from several human cell lines. Studying at single molecule resolution enables variation that are intrinsic to biological processes to be identified and therefore allow an accurate review of population distributions. The levels of miR-21 expressed at baseline in cell lines, MCF-10A, MCF7, MDA-MB-231, BEAS-2B, A549 and H1975 cells, were consistent with previous studies analysed by bulk measurements^27,64,69–71^. Notably, the heterogeneity within the cell populations was revealed within and between the cell lines. MiR-21 and miR-34a assays developed in this study were validated with miRNA mimics and antagomirs, and gold standard real-time qPCR. These miRNA assays can be easily adapted for other specific miRNA studies. When transitioning to scarce clinical samples, the practicality of the microfluidic chip is extremely advantageous as only 50 cells/experiment is required as opposed to real-time qPCR, which typically requires ~100,000 cells/experiment. In addition, we show that we can also detect low levels of miRNA in small quantities of biological fluid that may also be advantageous if cells are not available.

In summary, we demonstrated a complete lab-on-a-chip device that is sensitive to detect single molecules of miRNA in single cells via an extraction, label and amplification-free technique from primary human samples obtained non-invasively. Here, we showcase that our workflow can precisely and accurately analyse miRNAs in nasal fluid samples directly obtained from healthy volunteers. Healthy baseline single molecule count of miR-21 obtained from nasal fluid, CD3^+^ T-cells and EpCAM^+^ epithelial cells were quantified. The levels of miR-21 varied within and between patients for both nasal cells and fluid samples. This could be due to the rapid fluctuations in the environment of the nasal cavity in response to stimuli. Evidence of intra- and inter-individual variation of marker levels has been previously reported in nasal samples^56^.This workflow can be applied directly for single-cell analysis of many biological materials, especially for studying rare cell types that are extremely precious. One can perform a multiplex assay to detect and quantify more than one biomarker simultaneously from the same cell, for example miRNA and protein expressed from a single cell to improve accuracy and diagnostic value. Furthermore, the microfluidic chip device could be used for detection of targets expressed in several diseases, potentially enable generalised single screening test.

## Methods

### Probe and target miRNA oligonucleotides

All HPLC-purified oligonucleotides and synthetic miRNA sequences were purchased from Eurogentec (Eurogentec Ltd, Belgium) (Table 1).

**Table 1:** Synthetic miRNA sequences including one mismatch single base (highlighted in bold) and oligonucleotide sequences used for miR-21 and miR-34a detection assay.

	Sequence (5’–3’)
miR-21	UAGCUUAUCAGACUGAUGUUGA
miR-21 **1m**	**A**AGCUUAUCAGACUGAUGUUGA
miR-21 **22m**	UAGCUUAUCAGACUGAUGUUG**U**
miR-34a	UGGCAGUGUCUUAGCUGGUUGU
21-capture	NH_2_–C_12_-(TTTTTTTTTTTT)TCAACATCAGT
Biotin-21-capture	Biotinylated–C_12_-(TTTTTTTTTTTT)TCAACATCAGT
34a-capture	NH_2_–C_12_-(TTTTTTTTTTTT)ACAACCAGCTA
21-reporter-Cy5	CTGATAAGCTA-Cy5
34a-reporter-Cy5	AGACACTGCCA-Cy5
34a-reporter-Alexa488	AGACACTGCCA-Alexa488

### Chemical surface passivation and microarray

Chemical surface passivation was performed to minimise the non-specific binding of microRNA and aid the formation of the microarray printed capture spot size. Coverslips were prepared prior to passivation, a detailed cleaning protocol can be found in supplementary information. Two chemical passivation protocols were compared: (3-Aminopropyl)triethoxysilane (Sigma-Aldrich, UK) polyethylene glycol (Laysan Bio, USA) (APTES-PEG) and 3-Glycidyloxypropyl)trimethoxysilane (GPTS) functionalised surface. APTES-PEGylation procedure can be found in supplementary information.

GPTS passivation procedure was modified for miRNA detection. Pre-treated coverslips were rinsed with absolute ethanol and dried under nitrogen. Coverslips were plasma treated for 1 min to increase the surface energy and facilitate the functionalisation. GPTS solution was prepared with 100 mL absolute ethanol, 5 mL acetic acid and 2.5 mL GTPS under nitrogen. Coverslips were immediately immersed into a Coplin jar containing GPTS solution and incubated at room temperature in darkness for 10 min and sonicated for 1 min. This step was repeated twice. Coverslips were rinsed three times with absolute ethanol and sonicated for 15 min to remove unreacted GPTS. Coverslips were dried under nitrogen and immediately processed for microarray printing using an OmniGrid Micro microarrayer (Digilab, UK) and SMP2B printheads (ArrayIt, USA). A detailed microarray printing protocol detailed by Salehi-Reyhani et al.^67^. Printing solution was prepared at 1:1 ratio, 6 µL of 2.0 × 10^8^ molecules/nL capture probe with 6 µL printing buffer contained 6X saline sodium citrate (SSC; 20X; Thermo Fisher Scientific, UK) 3 M betaine (Sigma-Aldrich, UK). Capture spots were microarray printed at 40% humidity.

### Microfabrication

The microfluidic chip was designed using software (AutoCAD, Autodesk Inc.), SU-8 ‘master’ mould was fabricated using SU-8-based photolithographic lithographic technique explained in more detailed by Magness et al.^67^. Superstructure of the microfluidic chip was created by mixing 10:1 ratio of pre-polydimethylsiloxane (PDMS) polymer (VWR, UK) with curing agent (Sylgard 184 Silicon Elastomer Kit, Dow Corning), poured over ‘master’ wafer mould, degassed for 20 min and cured at room temperature for 48 hr. Once cured the PDMS chip were cut and peeled from the mould, the inlets and outlets were biopsy punched and drilled through the PDMS chip. To assemble the microfluidic chip device, the PDMS chip was oxygen plasma treated for 1 min and immediately assembled to the functionalised microarrayed coverslip. Analysis chambers of the PDMS chip were aligned to the microarrayed spots on the coverslip by translation with the aid of a custom-built 6-axis alignment rig.

### Optimisation of oligonucleotide probes

To determine the optimum microarray capture spot concentration, the capture probe was diluted with printing buffer to final concentration of 10^3^, 10^4^, 10^5^, 10^6^, 10^7^, 10^8^ and 10^9^ molecules/nL (Supplementary Fig. 3). Fluorescently tagged reporter probe concentration was optimised by performing a serial dilution in nuclease free water to achieve final concentration of 10^7^, 10^8^, 10^9^, 10^10^ and 10^11^ molecules/nL (Supplementary Fig. 3).

### Calibration procedure

To determine the absolute number of miR-21 and miR-34a molecules, both assays were calibrated individually to enable conversion from relative single molecule count to absolute number of target miRNA molecules. Hybridisation mixture was prepared with 5 µL of 6.02 × 10^9^ molecules/nL reporter probe, 100 µL of 20x SSC, 100 µL of 0.05% sodium dodecyl sulphate (SDS; Sigma-Aldrich, UK) and 100 µL of appropriate synthetic miRNA sequence concentration prepared by a serial dilution in nuclease free water (Thermo Fisher Scientific, UK) from 200 µM stock solution. This mixture was introduced into the chip and incubated in darkness for 2 h to allow the hybrids to form between the target miRNA and capture probe 1 × 10^8^ molecules/nL microarray printed inside the chambers. One chip was used as a control to determine the background noise, which only contained 21-reporter and hybridisation buffer.

### Cell culture

BEAS-2B cells were purchased from ATCC (Teddington, UK) and cultured in keratinocyte complete medium, containing 0.0317 mg/mL EGF Human Recombinant, 15.7 mg/mL Bovine Pituitary Extract. Human breast epithelial (MCF10A) cells were purchased from ATCC (Teddington, UK). A549 cells, MCF10A cells, MCF-7 cells and MDA-MB-231 cells were purchased from ATCC (Teddington, UK) and cultured in complete medium; (DMEM, Sigma-Aldrich, UK), 10% (^v^/_v_) FBS (BioSera, UK), 1% (^v^/_v_) L-glutamine (Sigma-Aldrich, UK), 1% (^v^/_v_) penicillin/streptomycin (Invitrogen, UK). HEK293 cells were purchased from ATCC (Teddington, UK) and cultured in complete medium (EMEM, 1X; Thermo Fisher Scientific, UK), 10% (^v^/_v_) FBS, 1% (^v^/_v_) L-glutamine. H1975 cells were cultured in Roswell Park Memorial Institute Medium (RPMI-1640; L-glutamine, phenol red, Sigma-Aldrich, UK), 10% (^v^/_v_) FBS, 1% (^v^/_v_) L-glutamine. Cells were incubated at 37 °C and 5% (^v^/_v_) CO_2_ and were passaged at a confluency of >80% within tissue culture flasks with a surface area of 25 cm^2^ (10 mL, Thermo Scientific, USA) using Accutase (BioLegend UK Ltd, UK) to detach cells. Cell lines were tested for mycoplasma contamination monthly using PCR methodology.

### Transfection

BEAS2B cells were nutrient starved for 24 h before transfection with mirVana miRNA 30 nM mimics (mirVana™ miRNA Mimic Negative Control #1, hsa-miR-21 MC10206, hsa-miR-34a MC11030) and 60 nM inhibitors (mirVana™ miRNA Inhibitor Negative Control #1, hsa-miR-21 MH10206, hsa-miR-34a MH11030) (Ambion, Life Technologies, Foster City, CA) using Lipofectamine RNAiMax (Life Technologies Ltd) for 24 h prior to experiment.

### Nasal sampling

This study was provided a favourable review and ethics approval was granted by Imperial College Research Ethics Committee (ICREC; reference: 18IC4779). Participants provided written informed consent and were recruited via word of mouth and were given a participant information sheet. All participants were aged over 18 years and recruited from the staff and students at Imperial College London. All participants were non-smokers or ex-smokers (for 10+ years). Those with underlying pulmonary/respiratory conditions (asthma, rhinitis, hay fever etc.) were excluded.

Each participant was sampled a maximum of 5 times. Sampling was obtained using Nasosorption^TM^ FX×I swab (Hunt Developments Ltd, Mucosal Diagnostics, UK). Synthetic absorptive matrix (SAM) device was placed inside lumen of the nostril, orientated to lie flat against the inferior turbinate, pressed onto nasal mucosa and held for 1 min to absorb mucosal lining fluid (MLF)^56^.

### Nasal sample processing

Once the sample has been collected, the foam of the matrix was removed from the applicator and suspended in 150 µL of RPMI-1640 (no phenol red; Corning) in an Eppendorf tube. This was gently vortexed to aid cell detachment. 150 µL Accutase was added and incubated at 37 °C and 5% (^v^/_v_) CO_2_ for 6 min. The matrix was further vortexed and clipped to side of tube to ensure there was no contact with the solution, then centrifuged at 700 x *g* for 5 min. Resulting supernatant was collected as nasal fluid and nasal cells were resuspended in 20 µL of RPMI-1640 (-) L-glutamine (no phenol red; Corning) with 5% (^v^/_v_) anti-human CD3 monoclonal antibody, Alexa Fluor®532 UCHT1 (ThermoFisher, UK) and 5% (v/v) anti-human CD326 (EpCAM) monoclonal antibody, Alexa Fluor®488 (ThermoFisher, UK). Nasal EpCAM^+^ epithelial cells and CD3^+^ T-cells were identified at wavelengths, *ʎ* = 488 and 532 nm and sorted in the microchannel of the microfluidic chip (Supplementary Fig. 7).

### Sample preparation

For single-cell experiments, 1 mL of cell solution was centrifuged at 300 x *g* for 5 min, the resulting supernatant was removed, and the remaining cell pellet was resuspended in 100 µL of RPMI-1640 (-) L-glutamine (no phenol red; Corning). Cell viability was performed using trypan blue exclusion prior to experiments.

### Single-cell experiment platform

The PDMS chip design consist of a main microchannel connected to 55 microchannels perpendicularly that leads to individual analysis chamber. Within the microfluidic chip device, 50 chambers are microarrayed with a miRNA capture spot on the surface of coverslip. Cell isolation, lysis and microRNA capture occurs within the analysis chamber (Supplementary Fig. 4).

The surface of the chip was passivated with 10 µL of 4% (^v^/_v_) PBSA, prepared with 4% (^w^/_v_) BSA (Fisher Scientific, UK) in DPBS (Sigma-Aldrich, UK) consisting of 10 µM of miRNA reporter probe (Eurogentec, Belgium). Cellular material with 4% (w/v) PBSA and 10 µM miRNA reporter probe were introduced into the inlet of the main microchannel. A syringe pump (LabSmith, USA) is attached to the outlet and set at a flow rate of 2 µL/min to pull the cellular material from the inlet to the outlet along the main microchannel. Once there were sufficient cells present in the main microchannel, the pump flow rate was set to 2 µL/min to enable cells to be trapped. All experiments were performed on an inverted microscope, Nikon TI-E (Nikon, Japan) using 60X, NA = 1.49 oil-immersion objective. Individual cells were isolated into the cubical within an analysis chamber (Supplementary Video 1), using an optical tweezer ytterbium fibre laser at 1070 nm (YLM-5, IPG, Photonics, UK) at a speed 5 µms^-1^. 80 µL of 4% (^v^/_v_) PBSA was introduced into the main microchannel to remove remaining excess cells. 20 µL of hybridisation mixture prepared with the same concentration as mentioned previously and was introduced into the main microchannel to diffuse into the chambers. The chip was incubated for 30 min in darkness, the presence of SDS in the buffer led to lysis of some cells. Dual lysis procedure was utilised as some cells that remained intact were optically lysed to ensure all the isolated cells were lysed. Cells were optically lysed by a single 6 ns pulse from Nd:YAG laser at 1064 nm (Sure-lite, SL I–10, Continuum, USA), which induces a cavitation bubble (Supplementary Video 2). The shear force expansion of the cavitation bubble eventually lyses the cell inside the cubical of the chamber mechanically. EM-CCD camera (IXON DU-897E; Andor Technologies, Ireland) was used to detect microRNA sandwich system via total internal reflection fluorescence (TIRF). TIRF operates such that only fluorescently tagged miRNA reporter within 200 nm above the surface of coverslip is scanned and imaged, thus once miRNA reporter is detected it is bound to captured target miRNA complex. The capture microarray spots were imaged using TIRF microscopy before and after cell lysis, then every 20 min for 2 h to allow equilibrium to be reached.

### Single-molecule count/readout and data analysis

All analysis of TIRF images was performed using FIJI program, which utilises GDSC Single Molecule Localisation Microscopy (SMLM) plugins developed by the University of Sussex^72^. Single molecules are observed on the capture spot as diffraction-limited intensity peaks and the images were categorised as congested or non-congested regime depending on the level of binding events. For non-congested regime, the diffraction-limited intensity peaks were individually resolvable and Gaussian peak fitting routine was used. Images that did not meet the tolerance criteria were rejected and the total single molecule count is the sum of all successful Gaussian fitted peaks. For congested images, peak fitting function is inappropriate due to the proximity of single molecules and instead the total intensity of image is divided by the mean single molecule intensity of the miRNA reporter probe.

### RNA extraction and real-time quantitative PCR

miRNAs were extracted using the miRNeasy kit (Qiagen) following the manufacturer’s protocol. Extracted RNAs were reverse-transcribed using the TaqMan normal MicroRNA Reverse Transcription Kit (Life Technologies). miRNA levels were detected by TaqMan MicroRNA Assays (hsa-miR-21-5p 002438, hsa-miR-34a-5p 000426) (Applied Biosystems, Life Technologies, Foster City, CA). RNU-48 (001006) (Thermo Scientific, USA), a small noncoding RNA, was detected as the endogenous control for miRNA detection. After the reactions, the Ct values were determined using fixed-threshold settings. Data were calculated using the 2^–ΔΔCt^ method of ΔΔCt = (Ct_Trarget_ – Ct_RNU-48_)X – (Ct_Trarget_ – Ct_RNU-48_) _Control_.

### Statistics and reproducibility

Data are expressed as individual points or mean ± SEM. Results were analysed using Mann–Whitney and Kruskal-Wallis tests, non-paired Student’s *t*-tests using GraphPad Prism 9.4 software (GraphPad Software, La Jolla, CA, USA). Values of *p* ≤ 0.05 were considered to be statistically significant.

### Reporting summary

Further information on research design is available in the Nature Portfolio Reporting Summary linked to this article.

## Supplementary information

Supplementary Information

Description of Additional Supplementary Files

Supplementary Video 1

Supplementary Video 2

Supplementary Data

Reporting summary

## Data Availability

Authors can confirm that all relevant data are included in the article and/or its supplementary information files. The raw data for the graphs and charts in the main figures is available as supplementary data and any remaining information can be obtained from the corresponding author upon reasonable request.
