# Supplementary material for: Single cell quantification of microRNA from small numbers of non-invasively sampled primary human cells
Source: Commun Biol. 2023 Apr 26;6:458. doi: 10.1038/s42003-023-04845-8 (PMC10133449; doi:10.1038/s42003-023-04845-8)
Supplement: Supplementary file 1 — Supplementary Information [file 42003_2023_4845_MOESM1_ESM.pdf]

Supporting information

Single cell quantification of microRNA from small numbers of non- invasively sampled primary human cells

Vanessa Ho[1-3], Jonathan R Baker[3], Keith R Willison[1,2], Peter J Barnes[3], Louise E Donnelly[3] and David R Klug[1,2]

[1]Institute of Chemical Biology, Molecular Sciences Research Hub, Imperial College London, 82 Wood Lane, White City, London, UK, W12 0BZ

[2]Department of Chemistry, Molecular Sciences Research Hub, Imperial College London, 82 Wood Lane, White City, London, UK, W12 0BZ

[3]National Heart and Lung Institute, Imperial College London, Guy Scadding Building, Dovehouse Street, London, UK, SW3 6LY

Supplementary methods

Microfluidic chip design

Various microfluidic chip designs were used depending on the purpose. To determine the optimal concentration of capture and reporter probes, an open chip with three or five wells was used to perform rapid probe screening. Standard microfluidic affinity capture chip consisted a main channel 35 mm x 1mm x 50 µm perpendicularly connected to fifty five chambers with dimensions of 300 x 300 x 50 µm and chamber volume of 4.5 nL. In each individual analysis chamber, a microRNA complimentary capture probe was microarrayed.

Glass coverslip preparation

The coverslips undergo an extensive cleaning pre-treatment to ensure the surface is free of any artefacts such as fluorescent contaminants that can lead to a high background interference and obscure single molecule binding. Glass coverslips with dimensions 24 x 50 x 1.5 mm were placed into a coplin jar and washed three times with MilliQ water. Coverslips were repeatedly rinsed with MilliQ water in between different solutions and sonicated in following solutions for different length of time: 20 min in 1M potassium hydroxide, 5 min in MilliQ water, 15 min in acetone, 20 min in 1M potassium hydroxide, 20 min absolute 100% ethanol. Coverslips were then dried under nitrogen gas and stored at 4°C until chemical passivation.

APTES-PEG surface passivation

Prior to silanisation process, coverslips were rinsed three times with MilliQ water and methanol, and further sonicated with methanol for 20 min. Coverslips were immersed into a solution of APTES prepared with 1 mL APTES, 100 mL methanol and 5 mL acetic acid, and incubated in darkness at room temperature for 10 min. Coverslips remained immersed in the APTES solution and were sonicated for 1 min, then further incubated in darkness at room temperature for 10 min. This enables the adsorption of a layer with alkoxylsilane molecules, coated with free amine groups on the glass surface. Coverslips were washed three times with methanol and MilliQ water to remove unreacted APTES, then dried under nitrogen.

PEGylation of the coverslips enabled the free amine groups to react covalently with PEG polymers that contained a Succinimidyl Valeric Acid (SVA) moiety. Coverslips were first functionalised in a prepared solution of mPEG/Biotin-PEG-SVA 5000 mW at saturating concentrations. This solution was prepared with 15 mg of Biotin-PEG-SVA and 50 mg of mPEG-SVA dissolved in 400 μL of 1 mM sodium bicarbonate solution. 80μL of this prepared solution transferred onto a coverslip and sandwiched to another coverslip. Sandwiched coverslip were placed into a humidified sterilin box to incubate in darkness for 2.5 h. Coverslips were rinsed with MilliQ water and dried under nitrogen.

Coverslips were further functionalised with NeutrAvidin® to faciliate the formation of covalent microarray capture probe on the glass surface. 90 μL of 1 mg/mL NeutrAvidin® in PBS solution was transferred onto PEG-biotinylated-coated coverslip and sandwiched to another coverslip. Sandwiched coverslip were placed into a humidified sterilin box to incubate in darkness for 1 h. Coverslips were rinsed with MilliQ water, then dried under nitrogen and immediately processed for microarray printing.

Supplementary figures

Open Chip

Wells: 4 mm x 8mm

10 microspots / row

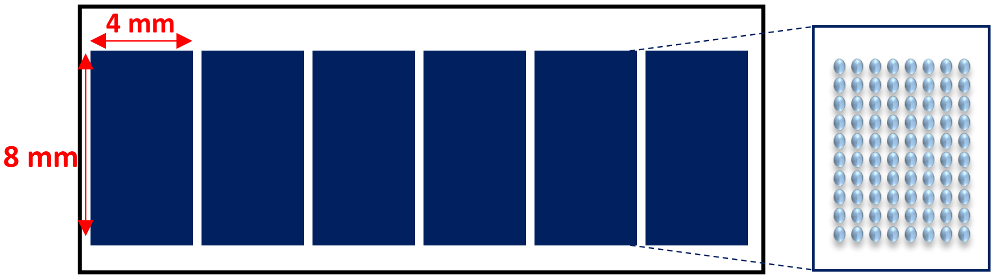

Standard Chip

Chamber: 300 µm x 300 µm x 50 µm (4.5 nL)

1 microspot / chamber

Cubicle: 40 µm x 40 µm x 50 µm

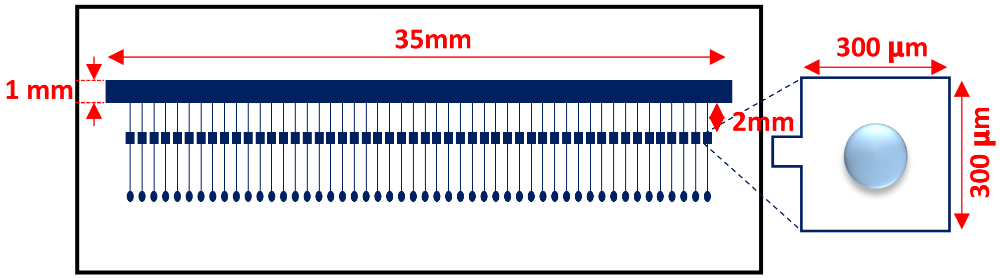

Supplementary Fig. 1: Microfluidic chip designs.

Designs were created using AutoCAD depending on the purpose. Open chip consist of 3-6 wells each well microarrayed with maximum of 80 microspots. Standard chip consist of a main microchannel (35 mm x 1mm) connected to 55 microchannels that leads to an individual chamber (300 µm x 300 µm x 50 µm) with a cubicle (40 µm x 40 µm x 50 µm). 50 of these chambers were microarrayed printed with one microspot.

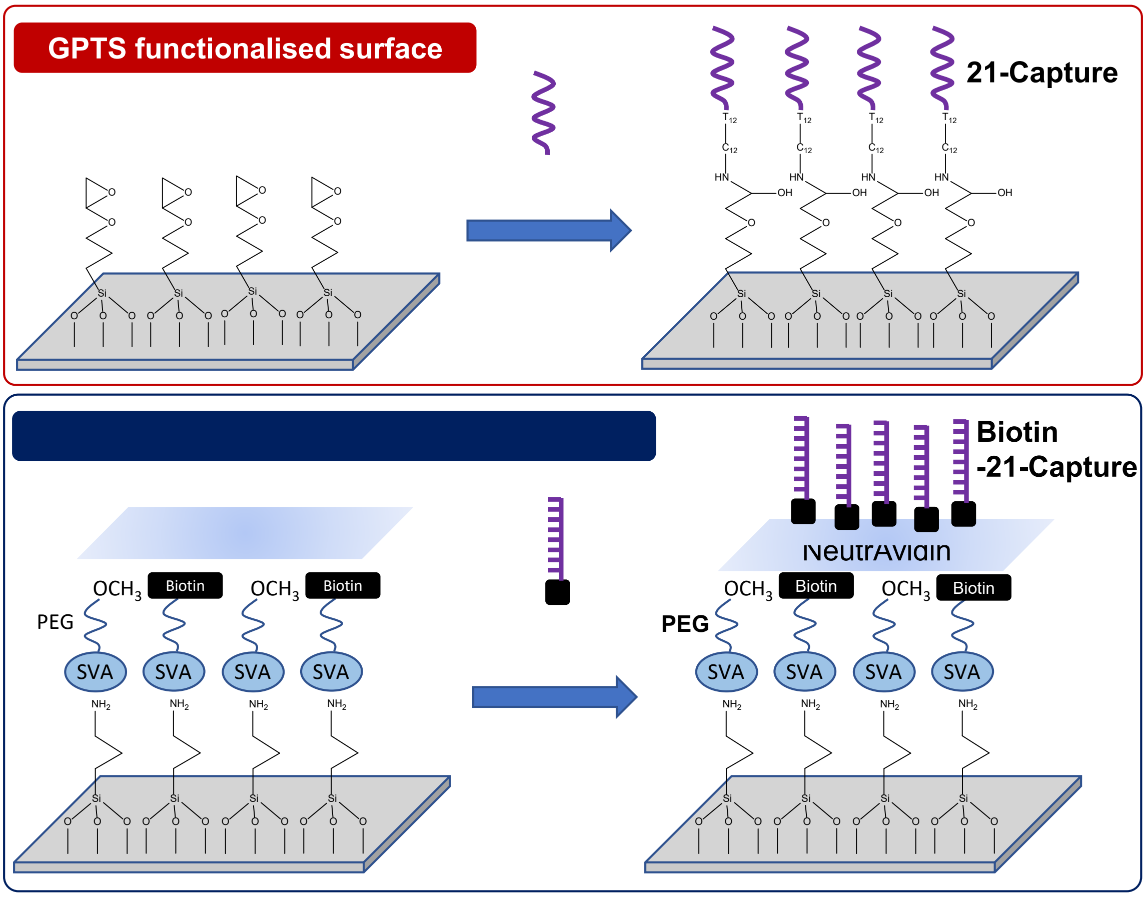

Supplementary Fig. 2: Schematic of chemical surface functionalisation

Schematic illustration of passivation on glass surface. GPTS passivated surface was microarrayed with 21-capture probe. Primary amine-epoxy addition occurs where the epoxide ring opens in presence of ethanol and amine group from the capture probe at room temperature. APTES-PEG passivated surface was microarrayed with biotin-21-capture probe. Free amine groups on the APTES surface reacts with mPEG/Biotin-PEG-SVA in presence of sodium bicarbonate. The surface is then coated with NeutrAvidin and the biotin group of capture probe is bound.

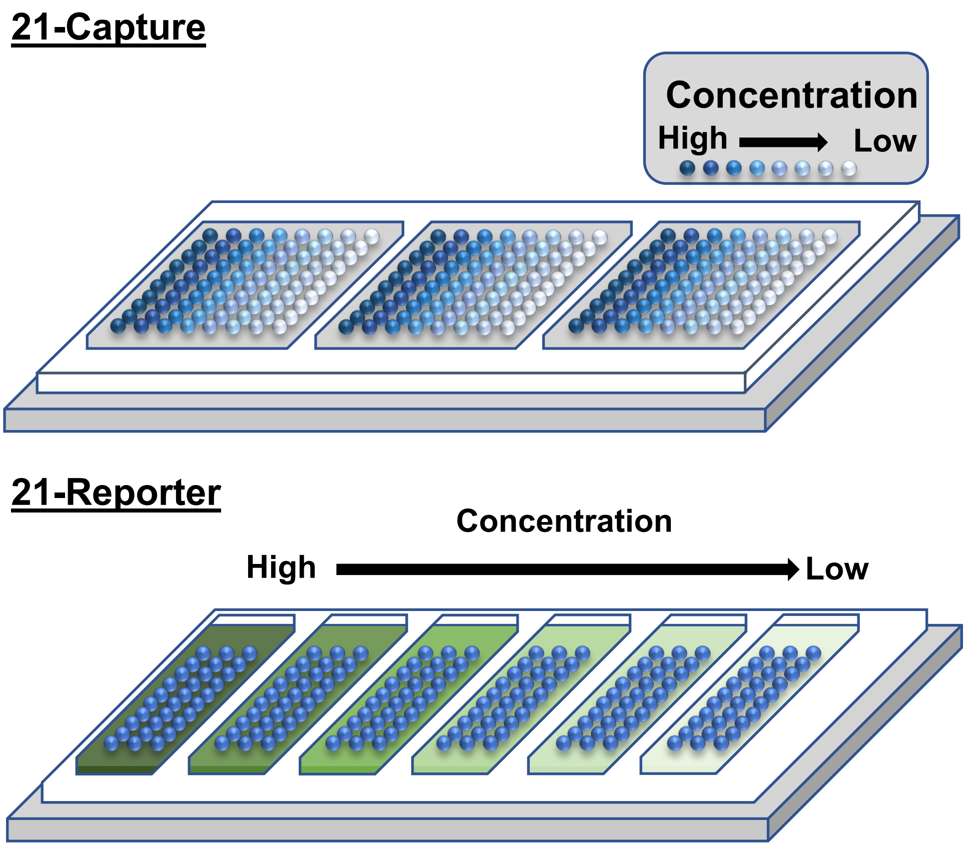

Supplementary Fig. 3: Schematic illustration of an open chip platform to screen different probe concentrations

21-capture open chip consist of three wells with eight rows per well and each row were microarray printed with fifteen capture spots at the same concentration. Different concentration of capture probe, 10^3-10^9 molecules/nL, were printed sequentially shown as different colour shade. 6.02 x 10^9 molecules/nL of reporter probe was introduced to second and third well. 21-reporter open chip contains six wells, each with forty-five capture, 1 x 10^{10} molecules/nL, microarray printed. Different concentrations of reporter probe were introduced into each well, 10^7-10^{11} molecules/nL represented as different colour shade. TIRF images were obtained with 10^6 molecules/nL of synthetic miR-21 in hybridisation buffer.

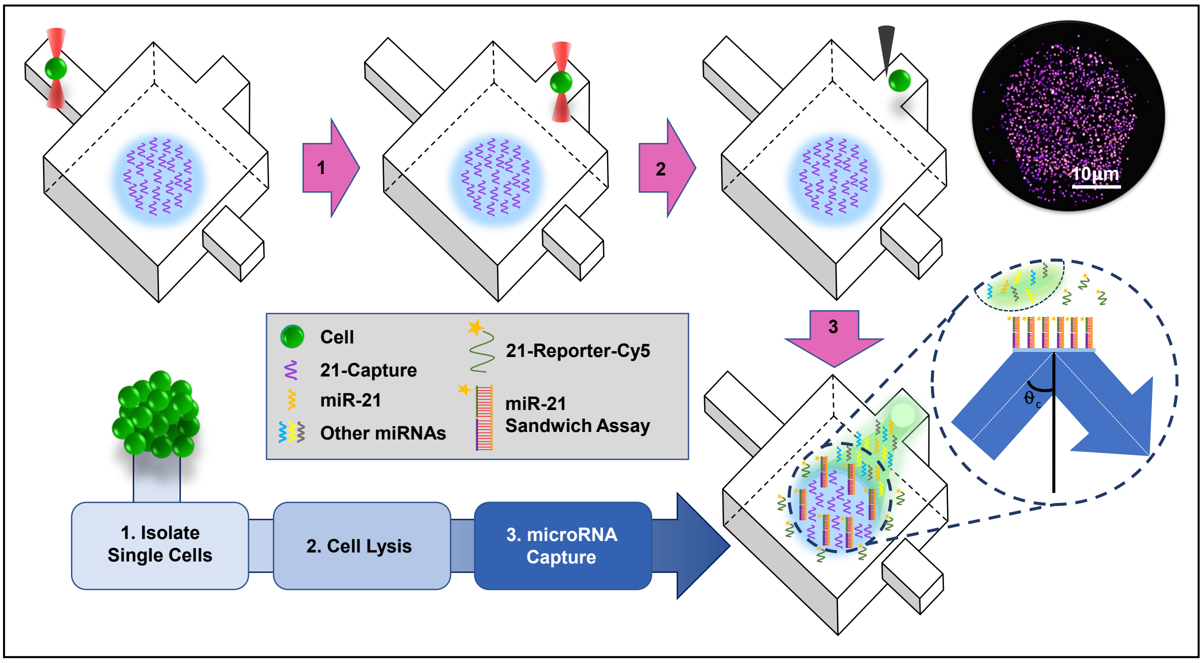

Supplementary Fig. 4: Schematic diagram of single cell experiment workflow

Three-step process indicated by the purple arrows. Individual cells are optically trapped into the cubicle of the chamber. Cells are lysed upon mechanical induced optical lysis within the chamber. This releases the intracellular components into the chamber. Target microRNA, miR-21 binds to the capture probe on microspot and fluorescently tagged reporter probe forming a sandwich assay and detected using total internal reflection fluorescence (TIRF) microscopy.

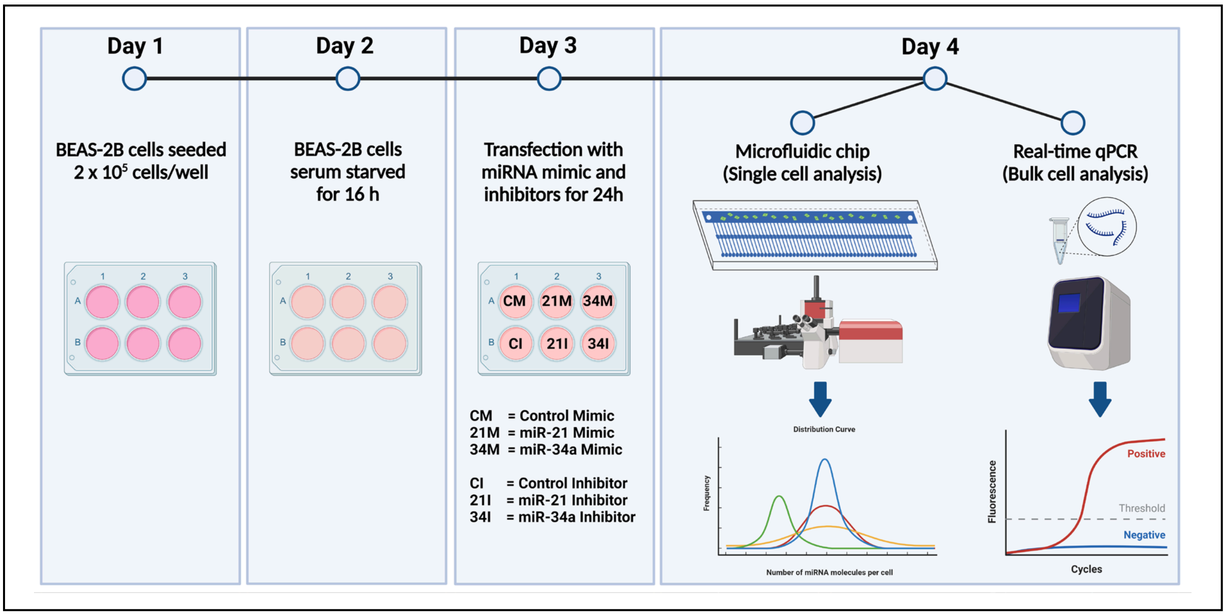

Supplementary Fig. 5: Timeline of miRNA assay validation using miRNA mimics and inhibitors.

BEAS-2B cells were seeded on a 6-well plate at 2 x 10^5 cells/well and left to adhere. Cells were serum starved for 16 h followed by transfection with miRNA mimics and inhibitors for 24 h. MicroRNA expression were measured by single cell analysis using microfluidic chip and real-time qPCR bulk cell analysis.

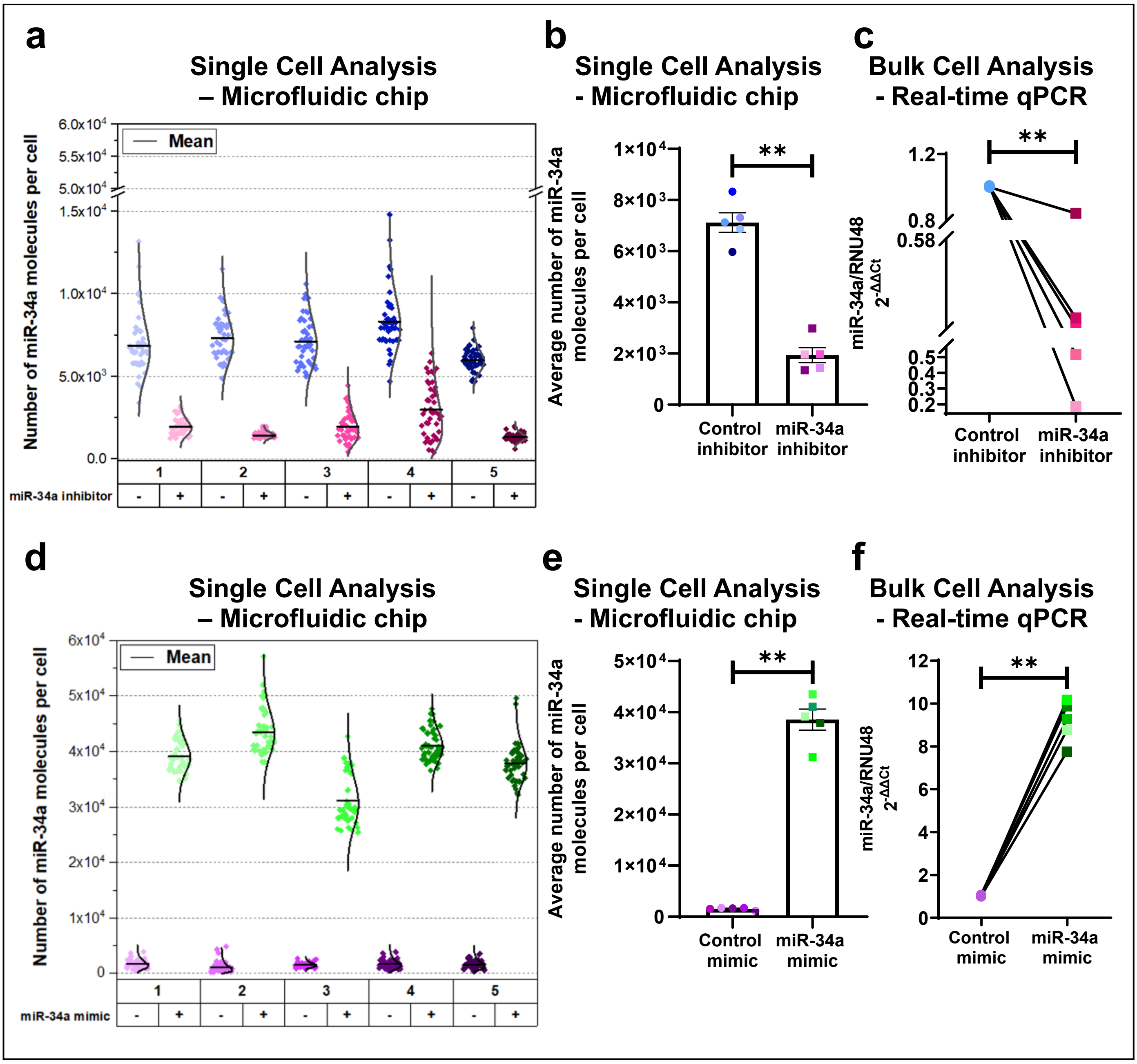

Supplementary Fig. 6: Validation of miR-34a assays using specific miR-34a mimics and inhibitors.

BEAS-2B cells were overexpressed with miR-34a mimic (Green), miR-34a antagomir (Pink), control mimic (Blue) or control inhibitor (Purple) for 24h and the expression of miR-34a was assessed using microfluidics and real-time qPCR (n=5). Panel **a)** Single cell distribution of miR-34a molecules expressed in single BEAS-2B cells transfected with inhibitor or control. Panel **b**) Comparison of average miR-34a expression between BEAS-2B cells transfected with inhibitor and control measured by single cell analysis. Panel **c)** MiR-34a expression in BEAS-2B cells transfected with inhibitor or control measured by real-time qPCR and normalised to RNU-48. Panel **d**) Distribution of miR-34a expression in BEAS-2B cells transfected with mimic or control measured in single cells. Panel **e**) Comparison of average miR-34a expression in single BEAS-2B cells transfected with mimic or control. Panel **f)** Analysis of miR-34a expression

in BEAS-2B cells transfected with mimic or control by real-time qPCR. The same shade of colour represents the same sample of BEAS-2B cells. The different shades represent experimental replicates. The black line represents a normal or gamma distribution fit to the data and the mean. Data were analysed using Mann-Whitney U test, **P < 0.01

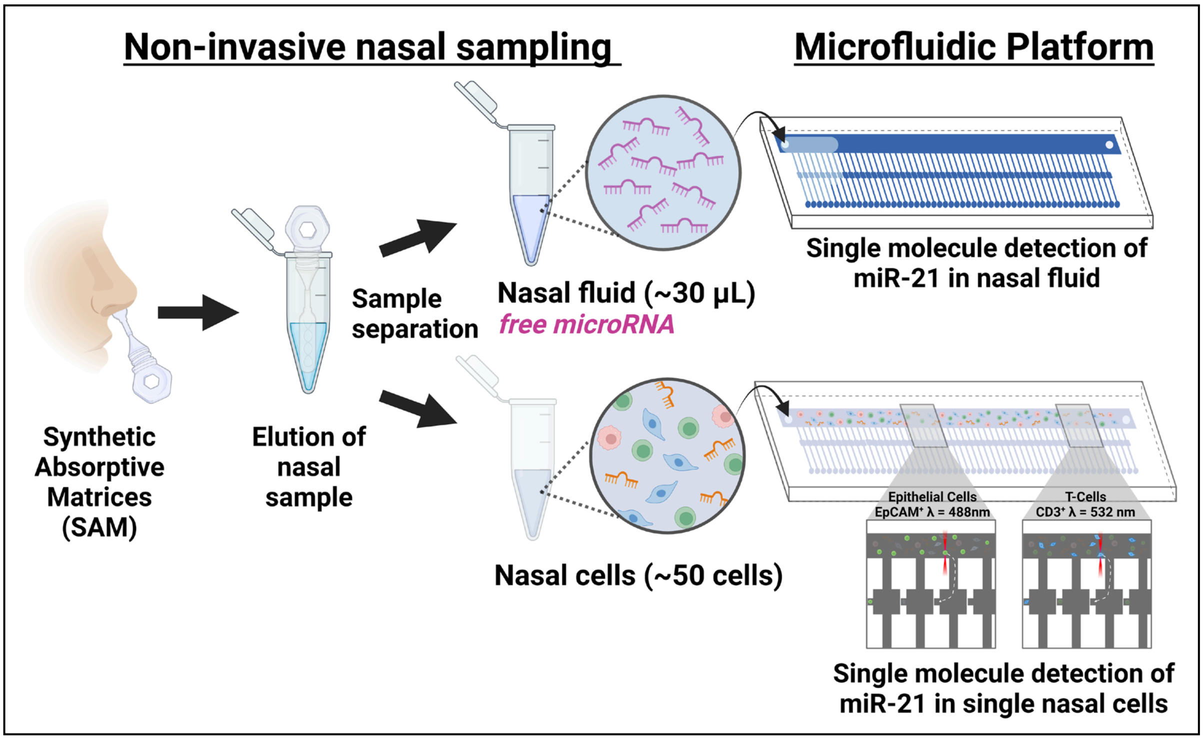

Supplementary Fig. 7: Schematic illustration of non-invasive sampling method and microfluidic platform

Nasal samples were obtained from subject's nose using synthetic absorptive matrices (SAM). Samples were eluted and separated in nasal fluid and nasal cells. MicroRNAs (miRNAs) expressed in nasal fluid (30 µL/experiment) and nasal cells (50 cells/experiment) were analysed using separate microfluidic chip devices. Cell surface labelled epithelial cells ($Epcam^{+}$) and $CD3^{+}$ T-cells were fluorescent at wavelengths, ʎ = 488 and 532 nm and sorted on-chip. Figure generated by Biorender

Supplementary Video 1: Video of optical trapping

Visualisation of a single BEAS-2B cell being transported along a microchannel into a cubicle of the analysis chamber using an optical tweezer. Video taken under brightfield.

Supplementary Video 2: Video of optical lysis

Visualisation of an isolated BEAS-2B cell being optically lysed within an analysis chamber by a single pulse from Nd:YAG laser at 1064 nm. Video taken under brightfield.
