# Supplementary material for: Single cell quantification of microRNA from small numbers of non-invasively sampled primary human cells
Source: Commun Biol. 2023 Apr 26;6:458. doi: 10.1038/s42003-023-04845-8 (PMC10133449; doi:10.1038/s42003-023-04845-8)
Supplement: Supplementary file 2 — Description of Additional Supplementary Files [file 42003_2023_4845_MOESM2_ESM.pdf]

# Description of Additional Supplementary Files

**File name:** Supplementary Video 1

**Description:** Video of optical trapping Visualisation of a single BEAS-2B cell being transported along a microchannel into a cubicle of the analysis chamber using an optical tweezer. Video taken under brightfield.

**File name:** Supplementary Video 2

**Description:** Video of optical lysis Visualisation of an isolated BEAS-2B cell being optically lysed within an analysis chamber by a single pulse from Nd:YAG laser at 1064 nm. Video taken under brightfield.

**File name:** Supplementary Data

**Description:** Data pertaining to the graphs in the figures
